# IDENTIFICATION OF ITEMS ASSESSING GENERATIVITY AND EGO INTEGRITY IN A LONGITUDINAL STUDY OF AGING

**DOI:** 10.1093/geroni/igae098.2609

**Published:** 2024-12-31

**Authors:** Olivia Duchow, Bryan James, Tomiko Yoneda

**Affiliations:** University of California Davis, Davis, California, United States; Rush University Medical Center, Chicago, Illinois, United States; University of California Davis, Davis, California, United States

## Abstract

Generativity, the seventh stage from Erikson’s psychosocial stages of development, is defined as the willingness to guide and inspire younger generations to promote generational wellbeing. The final stage, ego integrity, is characterized by an overall sense of life accomplishment and fulfillment. Although reminiscence therapy increases generativity and ego integrity, the extent to which these constructs are associated with health outcomes in older adulthood is unclear, as most work in this field relies on experimental designs and small samples. Thus, in the current work, we aim to develop a scale assessing generativity and ego integrity based on items administered to older adults in the Memory and Aging Project (MAP; N=~1700; Mage=65 years), which is a longitudinal study of older adults in the Chicago region with up to 24 annual measurement occasions until death. Development of this scale will permit follow-up investigation of the impact of generativity and ego integrity on long-term health outcomes, such as incident dementia diagnosis and mortality risk. Based on the existing literature, we identified numerous generativity and ego integrity scales (k=12). We then identified all potential items administered to MAP participants that may overlap with these items. Using artificial intelligence, we identified conceptually overlapping items to create a novel scale assessing generativity and ego integrity in MAP. We will report the discriminant validity, construct validity, and test-retest reliability of this scale.

